# Antifungal management and resource use in patients with acute myeloid leukaemia after chemotherapy – retrospective analysis of changes over 3 yr in a German hospital

**DOI:** 10.1111/j.1600-0609.2011.01704.x

**Published:** 2012-01

**Authors:** Angelika Böhme, Johannes Atta, Sabine Mousset, Birgit Ehlken, Margarita Shlaen, Gesine Bug, Hubert Serve, Dieter Hoelzer

**Affiliations:** 1 Department of Medicine, Hematology/Oncology, Goethe-UniversityFrankfurt/Main, Munich, Germany; 2 IMS HealthMunich, Germany

**Keywords:** acute myeloid leukaemia, antifungal management, chemotherapy, costs, posaconazole, antifungal prophylaxis

## Abstract

*Objectives:* To describe changes in costs of managing hospitalised patients with acute myeloid leukaemia (AML) after chemotherapy in Germany over 3 yr, with a special focus on prophylaxis and treatment patterns as well as resource use related to invasive fungal infections (IFI). *Methods:* The study was conducted as a retrospective, single-centre chart review in patients with AML hospitalised for chemotherapy, neutropenia and infections after myelosuppressive chemotherapy from January 2004 to December 2006 in Germany. The following resource utilisation data were collected: inpatient stay, mechanical ventilation, parenteral feeding, diagnostics, systemic antifungal medication and cost-intensive concomitant medication. Direct medical costs were calculated from hospital provider perspective. *Results:* A total of 471 episodes in 212 patients were included in the analysis. Occurrence of IFI decreased from 5.9% in 2004 to 1.9% in 2006. Mean (±standard deviation) hospital stay decreased from 28.7 ± 17.9 d in 2004 to 22.4 ± 11.8 d in 2006. From 2004 to 2006, the use of a single antifungal drug increased from 30.4% to 46.9%, whereas the use of multiple antifungal drugs decreased from 24.4% to 13.1%. The use of liposomal amphotericin B declined between 2004 and 2006 (21.4% vs. 3.8%) and caspofungin between 2005 and 2006 (19.3% vs. 8.1%). Total costs per episode declined from €19 051 ± 19 024 in 2004 to €13 531 ± 9260 in 2006; major reductions were observed in the use of antimycotics and blood products as well as length of hospital stay. *Conclusion:* Analysis of real-life data from one single centre in Germany demonstrated a change in antifungal management of patients with AML between 2004/2005 and 2006, accompanied by a decline in total costs.

Prolonged and profound neutropenia, defined as an absolute neutrophil count of <500/μL for more than ten consecutive days, is a common severe complication during remission induction chemotherapy of patients with acute myeloid leukaemia (AML) or myelodysplastic syndrome (MDS) (1). Invasive fungal infection (IFI) is a major cause of mortality in patients with neutropenia caused by a haematologic malignancy, its therapy or both. Fatality rates range from 60% to 90% and even non-fatal IFIs complicate or delay further chemotherapy, thereby impairing the treatment for the haematologic underlying disease (2, 3). The incidence of IFI has increased within the recent years, because of a rise in the number of immunocompromised patients (4–6). Reasons are supposed to be changes in the treatment for haematologic malignancies: formerly by more intensive cytotoxic treatment, nowadays by increasing number of elderly patients treated curatively and of transplantations with stem cells of unrelated or mismatch donors (5, 7). Early diagnosis of IFI is difficult as symptoms (e.g. fever and dyspnoea) are non-specific and untreated IFIs become rapidly fatal. Therefore, antifungal prophylaxis is a commonly used management strategy in patient populations with neutropenia (5, 8). Most IFIs in immunocompromised patients are caused by *Candida* species, but during the last years, the epidemiology of IFI changed. The adoption of antifungal prophylaxis has lead to a decrease in invasive candidiasis, in parallel with the occurrence of resistant *Candida* species and with an increase in infections by *Aspergillus* species and other filamentous fungi (9, 10).

Posaconazole, a novel broad-spectrum azole, has received approval by the European Medicines Agency (EMA) and by the US Food and Drug Administration (FDA) in 2006 (11). The efficacy of posaconazole as prophylaxis in high-risk patients has been shown to be superior to that of either fluconazole or itraconazole (12). Therefore, the European Conference on Infections in Leukemia recommends posaconazole for prophylaxis in allogeneic haematopoietic stem cell transplant recipients and patients receiving induction chemotherapy for acute leukaemia (5, 13).

In addition to increased morbidity and mortality of the affected patients, treatment for fungal infections is associated with significant costs for the healthcare system. The hospitalisation costs of patients with aspergillosis in 2009 were estimated to be, dependent on the underlying disease, between US $48 110 and US $80 468 higher than those of comparable patients without the infection (14, 15).

Budget constraints to hospitals are increasing over the time, and introduction of new substances or new indications resulting in extended use gives rise to objections about resource allocation and possible cost increase. This study addresses antifungal prophylaxis and treatment patterns as well as resource use related to the management of patients with AML after chemotherapy from hospital provider (HP) perspective in Germany from 2004 to 2006 with a special focus on prophylaxis and treatment for IFI. To provide real-life data for posaconazole, the analysis was carried out also for a subgroup of patients who had received posaconazole prophylaxis (PP) in 2006.

## Patients and methods

The study was conducted as a retrospective, single centre chart review in hospitalised AML patients after myelosuppressive chemotherapy from January 2004 to December 2006 in the University Hospital/Frankfurt Main, Germany. During the years 2005 and 2006, both chemotherapy regimens and antifungal management (except the posaconazole prophylaxis that was introduced in January 2006) had been nearly identical.

### Data collection

Information was obtained from medical records. All information was recorded onto structured data abstraction forms. Proven or probable IFI was diagnosed according to EORTC/MSG definitions (16). Resource utilisation was collected covering the complete hospital stay in patients hospitalised because of chemotherapy, neutropenia and/or infections. The admission date was considered as index date, and all data were collected from this point onward until the date of discharge. The following resource utilisation data were considered: inpatient stay (normal ward or ICU), mechanical ventilation, parenteral feeding, diagnostics, systemic antifungal medication, cost-intensive concomitant medication. Adverse events were collected and reported to Essex Pharma.

### Resource use and cost analysis

Direct medical costs per episode associated with above-mentioned resource utilisation were calculated from the HP perspective.

To evaluate costs, the quantity of each resource consumed was multiplied by the respective unit cost for each resource (e.g. price per tablet or injection, cost for hospital stay). Different cost data sources were used for calculation. Medications were multiplied by the prices according to the German pharmaceutical index ‘Rote Liste’ reduced by margins for hospital pharmacies (17). Unit cost for diagnostics was obtained from DKG-NT (18). Unit costs for hospital stay (without medication and procedures) were taken from a hospital internal database. Unit cost for transfusions was derived from manufacturers directly or from a pharmacy department of a university hospital.

The analyses were conducted for complete hospitalisation episodes, which mainly included the chemotherapy plus following neutropenic period plus infection complication. Until 2004, the patients were mainly hospitalised from start of chemotherapy until regeneration of neutrophils, but during 2004, the admission policy gradually began to change: a percentage of patients (about 10% each year) were discharged after the end of chemotherapy if clinically justified and re-admitted at the onset of neutropenia (neutrophil count <500/μL). In these cases, consisting of two hospital stays for the same chemotherapy episode resource consumption and related cost of the hospitalisations were added to achieve results for the episode as a whole.

Occurrence of IFI, antifungal management and total costs per episode were stratified by year of treatment (2004, 2005 and 2006). The costs for antifungals included also the antifungal prophylaxis. Total costs were also stratified by chemotherapy regimen for the subgroup of patients receiving PP compared with patients without PP. Based on the number of available episodes for analysis in the PP group, the following two chemotherapy regimens were selected for comparison: idarubicin/cytarabine/etoposide (regimen ICE; dosages for younger patients) ± valproic acid ± all-trans-retinoid acid and idarubicin/cytarabine/etoposide (regimen IDAV; dosages for elderly patients) (induction 1 or 2 each), which are described later in detail. Posaconazole prophylaxis was applied only in 2006. Patients without PP were pooled from the years 2005 and 2006, because treatment patterns in both years were quite similar (e.g. antifungal management).

### Cytostatic and supportive therapy regimens in patients with AML

During the study period, the patients had been treated with following chemotherapy first-line regimens:

#### Patients ≤60 yr

2004: SHG-Hannover-AML-1/99 (19): two induction cycles idarubicin/cytarabine/etoposide, two consolidation cycles with daunorubicin/cytarabine (1× intermediate dose, 1× high-dose: 2 × 3 g/qm day 1–6). For non-responders after first induction: one cycle fludarabine/idarubicin/cytarabine/granulocyte colony-stimulating factor (G-CSF) and one cycle high-dose cytarabine.

2005/2006: AMLSG 07/04 (20): similar induction cycles, less intensive consolidation with three cycles cytarabine 2 × 3 g/qm day 1, 3, 5; all cycles ± all-trans-retinoid acid and ± valproic acid. At the end of 2006, this protocol was changed and valproic acid was stopped.

#### Patients ≥61 yr

2004 until approximately October 2006: SHG-Frankfurt-AML-Elderly 1/99 (21): two cycles idarubicin/cytarabine/etoposide with G-CSF priming. First consolidation with fludarabine/reduced dosed idarubicin/intermediate dosed cytarabine, second consolidation with autologous stem cell transplantation if possible.

Since October 2006: AML-Sorafenib-phase II-Trial for Elderly (not published so far; ClinicalTrials.gov Identifier: NCT00373373:): one induction with daunorubicin/cytarabine, two consolidation cycles with cytarabine 2 g/qm day 1 + 3 + 5, all cycles ± sorafenib and sorafenib maintenance therapy for 1 yr.

The intensity of cytostatic relapse regimen did not markedly differ during 2004-2006. None of the study protocols excluded an antifungal prophylaxis with triazoles. Moreover, sorafenib has not shown any interactions with ketoconazole (22).

### Empirical and prophylactic antifungal therapy in persistent febrile neutropenia

Empirical antifungal therapy: In 2004, most patients received oral voriconazole or liposomal amphotericin B (L-AmB) 1–3 mg/kg. Owing to increasing fatal invasive mycoses in elderly patients, the empirical therapy was changed to liposomal AmB 3 mg/kg or caspofungin in 2005. This regime was continued during 2005 and 2006.

Prophylactic antifungal therapy: In 2004/2005, the patients did not receive any systemic antimycotic prophylaxis except secondary prophylaxis (mainly with voriconazole) in patients with prior invasive mycoses and further intensive chemotherapy. After presentation of study results by Cornely *et al.* (23) at the Annual Congress of the American Society of Hematology in 2005 (published in 2007), the prophylaxis with posaconazole in newly diagnosed AML and AML relapse induction cycles was established in January 2006.

### Statistics

Data were analysed by sas system (version 9.1) (SAS Institute Inc., Cary, NC, USA). For descriptive purposes, mean, median, standard deviation (SD), minimum and maximum were calculated for continuous variables and absolute and relative frequencies for categorical parameters. *T*-test and Wilcoxon two-sample test were used for the explorative comparison of continuous variables presented in two groups. Differences in distributions of continuous variables in more than two groups (e.g. evaluated parameters in three study observational years – 2004, 2005, 2006) were tested by using anova. The categorical variables such as percentage of patients with different disease status or gender distribution were tested exploratively with chi-square test. With respect to the exploratory character of analysis, no adjustment for multiple testing was undertaken.

## Results

**Figure 1:**
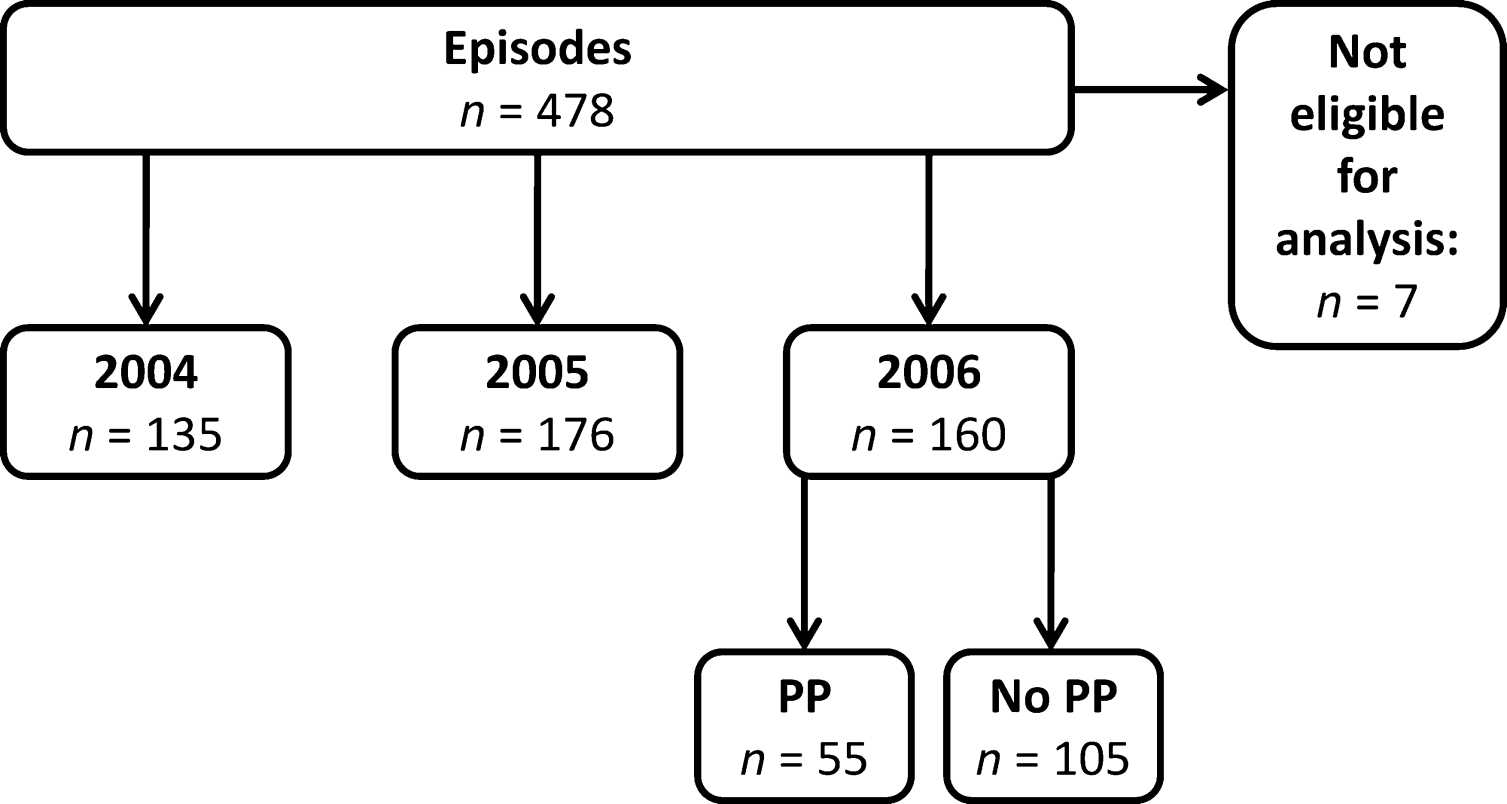
Disposition of hospitalisation episodes (PP, posaconazole prophylaxis).

In total, data for 478 episodes in 214 patients hospitalised during 2004–2006 were documented within 531 hospital stays. A total of 471 episodes in 212 patients were eligible for analysis (Fig. 1). Seven episodes were excluded because of the following reasons: four episodes (eight hospital stays) with PP during one hospital stay only; three episodes with PP and later treatment with posaconazole during the same hospital stay. In 2006, 55 patients received PP and 105 patients had not received PP because of other chemotherapy than induction, initially increased liver parameters, possibly problematical comedications. Demographic and clinical characteristics are summarised in Table 1. We found no significant differences in gender and de novo, secondary or relapsed AML over the 3 yr. Mean age differs significantly caused by the difference between 2005 and 2006, but did not result in a significant difference between the percentage of patients below and above 60 yr. The median duration of neutropenia (<500/μL) did not significantly change from 2004 to 2006. Significant differences have been remarked regarding the disease status with a lower percentage of patients with controlled AML in 2006. Differences over years observed in both patient groups treated with IDAV and ICE with respect to disease status and duration of neutropenia were not statistically significant. In patients receiving IDAV, median duration of neutropenia was significant lower than in patients receiving ICE (*P* = 0.0243).

**Table 1:** Demographic and clinical characteristics

	2004	2005	2006	*P*-value
Number of patients	83	77	80	
Mean age in years (SD)	59 (13)	57 (16)	63 (14)	0.0356
Percentage of patients >60 yr (in %)	59	51	68	>0.05
Sex distribution in % (male/female)	60/40	55/45	59/41	>0.05
Underlying disease *n* (%)^1^				
AML de novo	41 (49)	37 (48)	45 (56)	>0.05
AML secondary	34 (41)	36 (47)	27 (34)	
AML relapse	8 (10)	4 (5)	8 (10)	
Number of episodes	135	176	160	
Disease status *n* (%)^3^				
AML uncontrolled	37 (28)	31 (18)	49 (31)	0.0160
AML controlled	84 (62)	132 (75)	87 (54)	
Not available	14 (10)	13 (7)	24 (15)	
Median duration of neutropenia in days§	16 (0 – 79)	16 (0–75)	17 (0–71)	>0.05
Number of episodes – IDAV	30	31	21	
Disease status *n* (%)^3^				
AML uncontrolled	9 (30)	6 (19)	3 (14)	>0.05
AML controlled	18 (60)	25 (81)	17 (81)	
Not available	3 (10)	–	1 (5)	
Median duration of neutropenia in days§	19.5 (4–56)	16.0 (3–70)	15.0 (0–40)	>0.05
Number of episodes – ICE	7	43	24	
Disease status *n* (%)^3^				
AML uncontrolled	0 ()	4 (9)	4 (17)	>0.05
AML controlled	7 (100)	39 (91)	20 (83)	
Not available	–	–	–	
Median duration of neutropenia in days§	19 (12–23)	21 (0–38)	22.5 (10–71)	>0.05

AML, acute myeloid leukaemia.

*P* > 0.05: not significant.

1 At the first documented hospital stay.

§ Including patients without neutropenia.

3 At discharge.

Overall, probable or proven IFI was observed in 17 of 471 episodes (3.6%) (Table 2). Stratified by year, the occurrence of IFI decreased from 5.9% in 2004 to 1.9% in 2006. In patients with PP, no probable or proven IFI was documented.

**Table 2:** Occurrence of invasive fungal infections (IFI) (*n* = 471 hospitalisation episodes)

	2004 % of episodes (*n*) (*n* = 135)	2005 % of episodes (*n*) (*n* = 176)	2006 % of episodes (*n*) (*n* = 160)
IFI overall	5.9 (8)	3.4 (6)	1.9 (3)
Probable	1.5 (2)	1.7 (3)	1.3 (2)
Proven	4.4 (6)	1.7 (3)	0.6 (1)
IFI overall, by age group			
≤60 yr	3.2 (2)	2.8 (3)	1.6 (1)
>60 yr	8.3 (6)	4.4 (3)	2.0 (2)

The distribution of IFI by age group (combining 2004–2006) compared with the group without IFIs is not statistically significant (*P* = 0.2408; chi-square).

More IFIs were observed in patients >60 yr (11 of 17), but the result was not statistically significant because of small sample size.

The mean number of hospitalisation days decreased from 28,7 ± 17,9/29.3 ± 15.8 d in 2004/2005 to 22,4 ± 11.8 d in 2006. The differences between 2004/2005 and 2006 were statistically significant (*P* < 0.05).

The treatment patterns stratified by year in all eligible episodes (*n* = 471) are displayed in Table 3. With the exception of the use of imaging techniques (decline from 69.6% to 57.5% of patients) and growth factors (decline from 54.5% to 45.0% of patients), the treatment patterns were quite similar in 2004, 2005 and 2006.

**Table 3:** Treatment patterns (*n* = 471 hospitalisation episodes)

	Percentage of episodes in %		
	
	2004 (*n* = 135)	2005 (*n* = 176)	2006 (*n* = 160)
Hospital stay			
Haematology ward	100.0	98.9	99.4
ICU	4.4	6.8	7.5
Mechanical ventilation	1.5	3.4	3.8
Parenteral feeding	3.0	4.0	3.1
Diagnostic procedures			
Imaging techniques	69.6	64.2	57.5
Microbiological tests	80.7	84.1	85.6
Prophylaxis and treatment			
Antibiotics	86.7	85.8	83.8
Antimycotics	54.8	59.7	60.0
Virustatics	–	1.7	–
Growth factors	54.8	45.5	45.0
Blood products	94.8	91.5	94.4

ICU, intensive care unit.

From 2004 to 2006, the use of a single antifungal drug significantly increased from 30.4% to 46.9%, whereas the use of multiple antifungal drugs significantly decreased from 24.4% to 13.1% (Table 4). Single antifungal drug use was dominated by azoles (14.1%/12.5%) and increased from 23.7% in 2004 to 43.4% in 2006. Treatment with multiple antifungal drugs was dominated by the use of two drugs (two azoles, azole and L-AmB, azole and caspofungin). In 2004/2005 in 15.6%/15.3% of episodes, two antifungal drugs were applied, and in 2006 11.3%. The use of three or more antifungal drugs within one episode was necessary in 8.9% of episodes in 2004, 5.1% in 2005 and 1.9% in 2006. In patients with PP, in 76.4% of episodes, no further antifungal drug was necessary. In episodes with additional antifungal drug therapy (23.6%), especially an azole (10.9%) and caspofungin (7.3%) were administered.

**Table 4:** Distribution of antimycotics, stratified by year (*n* = 471 hospitalisation episodes)

	2004 % of episodes (*n*) (*n* = 135)	2005 % of episodes (*n*) (*n* = 176)	2006 % of episodes (*n*) (*n* = 160)	*P*-value
No antimycotics	45.2 (61)	40.3 (71)	40.0 (64)	>0.05
Single drug use	30.4 (41)	39.2 (69)	46.9 (75)	0.0153
Azole	23.7 (32)	29.6 (52)	43.4 (70)	
L-AmB	5.2 (7)	2.8 (5)	0 (0)	
Caspofungin	1.5 (2)	6.8 (12)	3.1 (5)	
Multiple drugs use	24.4 (33)	20.4 (36)	13.1 (21)	0.0408
Two azoles	6.7 (9)	2.3 (4)	5.0 (8)	
Azole/L-AmB	5.9 (8)	5.7 (10)	1.3 (2)	
Azole/caspofungin	1.5 (2)	5.7 (10)	4.4 (7)	
L-AmB/azole/caspofungin	4.5 (6)	4.0 (7)	0 (0)	
Two azoles/L-AmB	3.0 (4)	0 (0)	1.9 (3)	
Two azoles/caspofungin	0 (0)	1.1 (2)	0 (0)	
L-AmB/caspofungin	1.5 (2)	1.7 (3)	0.6 (1)	
L-AmB/two azoles/caspofungin	1.5 (2)	0 (0)	0 (0)	

L-AmB, liposomal amphotericin B.

When two, three or four antimycotics are listed, they were given sequentially, overlapping or as combination therapy in different formulations.

With the exception of posaconazole, the use of antimycotics declined from 2004 to 2006. The use of L-AmB declined from 21.4% to 3.8%, caspofungin from 19.3% in 2005 to 8.1%, fluconazole from 25.2% to 11.9% and voriconazole from 31.9% to 15.0%. This was accompanied with a reduction in the mean application period of L-AmB, caspofungin and voriconazole, but not observed for fluconazole (Table 5).

**Table 5:** Duration of application of antimycotics in days, stratified by year (*n* = 471 hospitalisation episodes)

	2004 (*n* = 135)		2005 (*n* = 176)		2006 (*n* = 160)	
			
	% of patients	Mean/SD in days	% of patients	Mean/SD in days	% of patients	Mean/SD in days
L-AmB (*n* = 29/25/6)	21.5	12.6/12.3	14.2	14.1/12.0	3.8	7.2/3.4
Caspofungin (*n* = 14/34/13)	10.4	14.8/15.7	19.3	19.4/15.4	8.1	12.2/6.6
Fluconazole (*n* = 34/45/19)	25.2	8.2/6.0	25.6	10.0/6.2	11.9	10.2/6.8
Itraconazole (*n* = 1/1/0)	0.7	–1	0.6	–1	0.0	–
Posaconazole (*n* = 0/0/58)	0.0	–	0.0	–	36.3	16.3/7.2
Voriconazole (*n* = 43/42/24)	31.9	13.2/11.3	23.9	14.2/10.6	15.0	9.1/10.1

L-AmB, liposomal amphotericin B.

1 Value for one patient not shown.

Overall, total costs amounted to €17 352 ± 15 971 per episode. The main cost drivers were hospital stay (42.1%), blood products (20.0%) and antimycotics (16.4%) followed by growth factors (10.1%) and antibiotics (8.1%).

**Figure 2:**
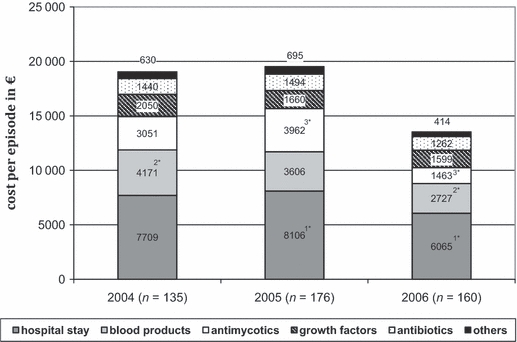
Mean direct medical costs per hospitalisation episode in € stratified by year of admission to the hospital (others: parenteral feeding, ventilation, diagnostics, virustatics and other drugs). Statistical significant differences: 1*P = 0.0099; 2*P = 0.0147; 3*P = 0.005).

Total costs per episode declined from €19 051 ± 19 024/€19 523 ± 17 599 in 2004/2005 to €13 531 ± 9260 in 2006. The difference between 2004/2005 and 2006 was statistically significant (*P* < 0.05). The main reduction was observed in the use of antimycotics (from €3051 ± 6271/€3962 ± 7806 in 2004/2005 to €1463 ± 2688 in 2006) and in hospital stay (from €7709 ± 5429/€8106 ± 5425 in 2004/2005 to €6065 ± 3254 in 2006) (Fig. 2). Both differences were statistically significant (*P* < 0.05).

**Figure 3:**
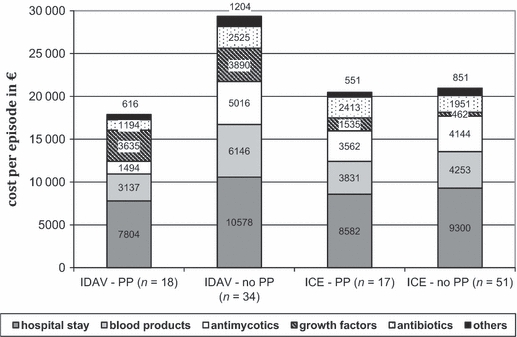
Mean direct medical costs per hospitalisation episode in € stratified by type of antifungal prophylaxis in patients receiving ICE regimen (idarubicin/cytarabine/etoposide; dosage for younger patients) ± valproic acid ± all-trans-retinoid acid or IDAV regimen (idarubicin/cytarabine/etoposide; dosage for elderly patients); others: parenteral feeding, ventilation, diagnostics, virustatics and other drugs; PP, posaconazole prophylaxis.

The stratification of costs was also conducted for patients with and without PP (Fig. 3). In patients after IDAV chemotherapy, mean total costs were significantly lower in patients receiving PP than in patients without PP (€17 880 ± 5059 vs. €29 359 ± 25 777; *P* = 0.0166). In patients after ICE chemotherapy, mean total costs were comparable (with PP: €20 474 ± 7510; without PP: €20 961 ± 13 433; *P* = 0.8533). In patients after IDAV chemotherapy, costs for antimycotics were significantly lower in patients receiving PP (€1494 vs. €5016; *P* = 0.0202). In patients receiving ICE chemotherapy, costs for antimycotics were quite comparable (€3562 vs. €4144; *P* = 0.6556).

## Discussion

Presented analyses provide first real-life data on the management of patients with AML after chemotherapy in a German University hospital over a time period of 3 yr with a special focus on antifungal treatment. These analyses are important to reflect the impact of new strategies in patient management on outcomes and cost.

Our data indicate a decline in the length of hospital stay from 2004/2005 to 2006 together with a considerable decline in total costs per patient episode. The length of hospital stay depends markedly on the intensity of chemotherapy regimens and duration of neutropenia, which is one of the most important risk factors for severe infections. Despite the attenuation of chemotherapy intensity, for example in consolidation therapy, especially by cytarabine dose reduction (see patients and methods), the duration of neutropenia did not differ from 2004 to 2006. A tendency to higher percentage of patients with uncontrolled leukaemia found in 2006 was possibly related to higher number of elderly patients. So, two main risk factors for prolonged hospital stay and occurrence of IFI have *not* decreased over the years. This is a crucial point for the assessment of the impact of posaconazole on resource use and associated cost. In contrast, the lower rate of IFI in 2006 may be a cause for shorter hospital stay. Besides the hospital stay, the trend towards lower cost was especially high in antimycotics and blood products.

During the last years, general efforts have been made to develop evidence-based strategies and recommendations for antifungal prophylaxis and treatment in patients with haematologic malignancies considering the epidemiological change to invasive aspergillosis (5, 8, 10, 24, 25). The higher costs for antifungal therapy in 2005 are caused by the change of empirical antifungal treatment preferring cost-intensive antimycotics like L-AmB and caspofungin. In 2006, both substances were less frequently used as well as applicated over a shorter time period than in 2005. These change resulted in a decline of antimycotic cost. The reduced use of L-AmB and caspofungin in 2006 may be interpreted as a consequence of an effective antifungal prophylaxis with posaconazole. Other causes, however, may have contributed to reduction in fungal infections and use of antifungal medication, e.g. AML remission rates or duration of neutropenia. But the comparison of neutropenia periods did not show statistically significant differences during the years, and the percentage of episodes with controlled leukaemia was significantly lower in 2006 compared with 2005. The inpatient exposure to aspergillus spores (air, bathroom/showers and other) may have differed also, but this factor is in general difficult to address. The increased use of a single drug regimen in 2006 may also be assessed as an indirect parameter for effective antifungal prophylaxis with lower rate of empirical and targeted antifungal treatment.

No probable or proven IFI was documented in the total PP group, and only in 13 of 55 episodes, additional antimycotics were given. This finding has also to be discussed with respect to aforementioned changes in cytostatic treatment, which may have contributed to a lower incidence of invasive mycoses in 2006. To circumvent the impact of that factor, we compared a small subgroup of 55 patients in induction therapy of AML (IDAV or ICE) receiving PP with patients undergoing the same chemotherapy regimens, but without PP. The empirical antifungal regimen was the same for both groups. The introduction of PP did not lead to an increase of costs. In contrast, for patients after IDAV chemotherapy, total costs per episode were considerably lower in patients with PP than in patients without PP. For the younger patients after ICE induction, however, total costs were quite similar in the PP group compared with the group without PP. That is astonishing, because the duration of neutropenia in ICE episodes was markedly longer, so a higher rate of IFI would have to be expected. In elderly patients, however, a generally higher risk of incidence and morbidity of IFI have been described (26, 27), which may be correlated with more extensive comorbidities. Also, the higher number of AML elderly patients with high-risk cytogenetics and lower remission rate may play a role (28). Also in our complete patient population, a tendency to higher number of IFI in elderly patients could be found, but the difference was not statistically significant. In summary, the introduction of PP did not result in higher costs. In elderly patient with AML induction therapy with IDAV, the PP was cost-saving.

In general, a comparison between healthcare costs generated in different countries has to be assessed carefully because of the differences of the health systems and charges for utilised resources.

Comparable data on total medical costs are available for the treatment of invasive aspergillosis in patients with haematologic malignancies. In a study conducted in the Netherlands on 269 patients, 12% had possible and 18% had probable or proven invasive aspergillosis. Mean medical costs per patient corrected for duration of neutropenia amounted to €21 130, €29 490 and €36 410 (costs for 2007) for patients without, with possible and with probable or proven invasive aspergillosis, respectively (29). Cost data from the USA arise to total hospital charges of US $191 974 ± 163 535 and US $45 064 ± 69 220 (costs for 2003) for patients with haematologic malignancies with or without aspergillosis, respectively (30). Taking the relatively low rate of IFI (4%) in our study into account, presented cost data are considerably lower than the costs evaluated for 2003 in the USA and somewhat lower than average cost without IFI in the Netherlands.

Our presented cost data of nearly €20 000 per episode in 2004/2005 correspond to data reported in a single centre bottom-up study from Germany. The direct costs in patients with AML for hospitalisation because of persistent fever and neutropenia amounted to €19 039 (95% confidence interval: €17 050–€21 029; costs for 2002) (31).

Many attempts have been undergone to save costs in the antimycotic treatment. In a multicentre randomised trial on high-risk, febrile, neutropenic patients with haematologic malignancies in France, the researchers found decreased costs of antifungal therapy by 35% for pre-emptive compared with empirical treatment (32). Pre-emptive treatment was defined as treatment of patients who had not only persistent or recurrent fever, but additional clinical, imaging or laboratory signs of fungal disease. First-line antifungal treatment was amphotericin B deoxycholate or L-AmB. In the pre-emptive group, 9.1% of patients had probable or proven IFI, whereas in the empirical group, only 2.7% (32). Mean costs for antifungal drugs amounted to €2252 SD €4050 in the empirical treatment group and to €1475 SD €3329 in the pre-emptive treatment group (costs for 2005). If L-AmB was used instead of amphotericin B deoxycholate, estimated costs were €4261 ± 4760 and €2509 ± 4099 in both groups, respectively. The declining average costs for antimycotics from €3051/€3962 in 2004/2005 to €1463 in 2006 are therefore in line with the data from the literature and reflect a general change in treatment strategies. It should be mentioned that more proven invasive IFIs were observed in the pre-emptive treatment group, and the authors concluded that ‘empirical treatment may provide better survival rates for patients receiving induction chemotherapy’.

According to a retrospective chart analysis in United States, the introduction of posaconazole did not lead to a change in the use or costs for antimycotics, antibiotics or total medications in the same patient setting (33). In contrast, results of decision analytic modelling on the cost-effectiveness of posaconazole in comparison with standard prophylaxis (itraconazole or fluconazole) suggest cost – saving potential of posaconazole in different European countries and United States (11, 34, 35). In about 53–87% of model population, prophylaxis with posaconazole leads to lower cost per patient with respect to costs of prophylaxis and IFI treatment than in the comparator group.

Further studies would be necessary to demonstrate the effectiveness of posaconazole prophylaxis in comparison with other prophylactic treatment regimens under real-life conditions in equivalent patient groups. But study results always depend on the hospital-specific risk factors, especially risk for aspergillosis (construction activities, hygienic conditions on the wards, air filtration and more) and especially the empirical treatment regimens. In multicentre studies, these effects may be levelled and the results may have no consequences for single hospitals. Cost-effectiveness of PP may be given in hospitals with higher rate of aspergillosis, internal recommendations preferring rigorous empirical therapy without waiting for given criteria for pre-emptive start and high use of expensive antifungal therapies. According to our experiences, a higher IFI risk in elderly patients with AML may be considered even if they are treated with lower intensive chemotherapy regimes. Meanwhile, it is important to consider also the gain in quality of life, which is given not only by reduced mortality but also by reduced morbidity.

All these aspects and the fact that posaconazole is recommended for antifungal prophylaxis in AML induction therapy with the highest level strength of recommendation and quality of evidence in national as well as international guidelines may complicate the decision for or against posaconazole prophylaxis (8). Hospital- and patient-specific risk factors should be decisive.

Some limitations of our study have to be mentioned. The study was conducted retrospectively in a single centre. The impact of PP on the occurrence of IFI was shown descriptively, but could not be assessed with respect to an equivalent matched comparator group. The patient numbers of the analysed subgroups with induction treatment ICE and IDAV are too small to provide concluding results, but may serve as a starting point for further research.

In conclusion, our real-life data from one single centre in Germany demonstrate a change in antifungal management of AML/MDS patients between 2004/2005 and 2006, accompanied by a decline in total costs. Reduced use of multiple antifungal drugs and decreasing antifungal therapies after introduction of posaconazole prophylaxis in 2006 could be demonstrated. Additional research is necessary to further improve antifungal strategies and to update cost development continuously.
